# Remission of OCD and ulcerative colitis with a ketogenic diet: Case Report

**DOI:** 10.3389/fpsyt.2025.1541414

**Published:** 2025-04-03

**Authors:** Lori Calabrese

**Affiliations:** ^1^ Touchpoints180™, South Windsor, CT, United States; ^2^ Innovative Psychiatry, LLC, South Windsor, CT, United States

**Keywords:** ketogenic diet (KD), obsessive-compulsive disorder (OCD), anxiety, ulcerative colitis (UC), inflammatory bowel disease, inflammation, metabolic disorders, ketogenic metabolic therapy (KMT)

## Abstract

**Background:**

There is little research describing the clinical use of a ketogenic diet in obsessive-compulsive disorder (OCD) or inflammatory bowel disease. We describe the first clinical application of a ketogenic diet in adult OCD with ulcerative colitis (UC) resulting in complete remission of OCD, clinical remission of UC, and improved metabolic health.

**Methods:**

A 37-year-old obese woman with longstanding OCD and ulcerative colitis was treated for 12 weeks with a personalized whole-food ketogenic diet (KMT 1:5:1 ratio) in a specialized metabolic psychiatry clinic. Adherence was assessed by capillary beta-hydroxybutyrate (BHB) and photojournaling of food intake. Remission of OCD was assessed by the Yale-Brown Obsessive Compulsive Scale (Y-BOCS), Florida Obsessive Compulsive Inventory scale (FOCI), and Clinical Global Impression-Improvement/Severity scale (CGI-S/I). Patient Health Questionnaire-9 (PHQ-9) and Yale Food Addictions Scale 2.0 (YFAS 2.0) assessed depression and food addiction. Remission of UC was assessed by the Partial Mayo Score (PMS) and the Ulcerative Colitis Patient-Reported Outcome (UC-PRO). Metabolic health was assessed by laboratories and bioimpedance. Quality of life was assessed using validated scales for flourishing, resilience, self-compassion, and subjective narrative.

**Results:**

Clinical remission of UC occurred within 3 weeks (PMS 0, UC-PRO 0). Progressive improvement in OCD was inversely related to oscillating BHB, with FOCI 0 at 9 weeks, and complete remission at 12 weeks (Y-BOCS 0, CGI-S 1). Body weight decreased 12.2%, with significant decreases in the percentage of body fat and visceral fat. Flourishing, resilience, and self-compassion improved 2- to 20-fold.

**Conclusion:**

Complete remission of OCD, clinical remission of UC, and marked improvement in metabolic health occurred within 12 weeks using a well-formulated personalized ketogenic diet (KMT ratio 1:5:1) with a meaningful positive impact on quality of life and significant improvements in flourishing, resilience, and self-compassion.

## Introduction

Obsessive compulsive disorder (OCD), the key example of obsessive-compulsive and related disorders (OCRDs), is a complex neuropsychiatric disorder affecting 2%–4% of individuals and 16.9% of postpartum mothers (1) that is often underrecognized and undertreated (2). It is characterized by recurrent, intrusive, unwanted thoughts and behaviors, which are often chronic, disabling, and accompanied by shame, disgust, or a need for “just right.” (3). OCD markedly interferes with quality of life (QoL), particularly in women (4, 5); symptom severity and comorbid depression are predictors of decreased QoL (6). It is highly comorbid with other OCRDs, with high lifetime histories of depression (84.7%), generalized anxiety disorder (71.5%), and social anxiety (47.9%) (7).

First-line treatments are cognitive behavioral therapy/exposure response prevention (CBT/ERP) and pharmacotherapy with a serotonin reuptake inhibitor (2) but are often underutilized, burdensome, or unavailable; only 40%–60% achieve partial response (8). Second-line or augmentation strategies for treatment resistance include dopamine antagonists and glutamatergic agents (9), but these are effective in less than half of serotonin reuptake inhibitor (SRI)-resistant OCD (3). Treatments specifically targeting the cortico-striatal-thalamo-cortical (CSTC) circuits include neuromodulation and, in severe cases, deep brain stimulation and cingulotomy (10).

Strong emerging evidence suggests that inflammatory dysregulation of the CSTC circuit underlies the pathophysiology of OCD (4), and modulation of this circuit by reducing immune and inflammatory microglial activation may offer an opportunity to directly target molecular mechanisms. However, despite strong links to inflammation, there is very little evidence for effective anti-inflammatory treatments in OCD (9).

OCD is associated with a 1.83 increased risk of metabolic syndrome (MetS), obesity, type 2 diabetes (T2D), and cardiovascular disease (11). A recent cross-sectional study reported the prevalence of MetS among adults with OCD was 39.2%; central adiposity was the most frequent component of MetS (68.2%) (12). Recent research supports a bidirectional relationship between OCD and T2D with impaired insulin sensitivity, induction of the inflammatory response, and insulin resistance (13).

Ulcerative colitis (UC) is a chronic, progressive, immune-mediated inflammatory bowel disease (IBD) affecting approximately 5 million people worldwide (14), with a dramatically increased prevalence in newly westernizing nations associated with increased dietary consumption of sugar and ultra-processed foods (15, 16). Recent pilot and cohort studies correlate the consumption of excess sugar, PUFAs, and emulsifiers to IBD and UC through alterations of the gut microbiome and disruption of gut epithelial cell barrier functions (17, 18).

UC is characterized by frequent bloody diarrhea, pain, fatigue, and progression, and more than 1/3 of patients require hospitalization or surgery. Comorbid anxiety and depression are as high as 41.8% and 62.3%, respectively, and double healthcare costs for UC patients (19). UC can profoundly affect QoL and is associated with a reduced life expectancy of 5 years (20).

Dietary interventions disappoint. Metanalyses of a wide range of dietary interventions for UC are hampered by low certainty data and insufficient evidence of benefit (21, 22), and a recent comprehensive review underscored this (23). Despite advances in medications, immunosuppressives, biologics, small molecules, and surgery, response varies widely, clinical remission can be fleeting, and complete remission is rare (24).

A therapeutic ketogenic diet (KD), known as a ketogenic metabolic therapy (KMT), offers the potential to directly improve dysregulated enteric permeability, reduce enteric inflammation and activation of the NLRP3 inflammasome, improve mitochondrial function, decrease oxidative stress and the release of inflammatory cytokines, modulate microglial neuroinflammation, and regulate neurotransmitter function, all potentially synchronizing to calm overactivity in the CSTC circuit (25). Although a recent case series describes the implementation and use of KMT in remission of major depression and generalized anxiety disorder (26), there are no published data regarding the clinical application of KMT in OCD, or particularly, OCD comorbid with systemic inflammation and autoimmune disorders.

Here we present a case demonstrating KMT as a novel treatment for OCD, leveraging the profound anti-inflammatory effects of a whole-food ketogenic diet to concurrently target and suppress OCD, UC, and autoimmune conditions in an adult with a 25-year history of OCD, 5-year history of moderately severe UC, psoriasis, Hashimoto’s thyroiditis, obesity, and central adiposity. We describe the evaluation and 12-week course of treatment, correlating the capillary beta-hydroxybutyrate/glucose ketone index (BHB/GKI) with time to remission and improvements in metabolic health and QoL.

## Case presentation

Our case is a 37-year-old employed married woman with a lifelong history of generalized anxiety, mild social anxiety, and intermittent skin-picking, who viewed anxiety as troublesome but a useful motivator. OCD began at age 10 and was unrecognized and persistent, with varying severity. She developed intermittent depressive episodes in her 20s, sought psychiatric evaluation, and was treated with psychotherapy with some relief. Exercise helped her manage stress. Anxiety increased during pregnancy and she experienced moderately severe postpartum depression, which improved spontaneously after many weeks. Months later, she began sertraline 50 mg daily with partial response and erratic adherence. During another pregnancy, she developed new-onset bloody diarrhea, progressing insidiously to multiple daily bloody diarrheal stools with cramping pain. Symptoms were attributed to pregnancy and hemorrhoids, which delayed referral and histopathologic confirmation of UC for more than a year. After delivery, harm OCD emerged, focused on the baby, escalating dramatically to consume 75% of her thoughts, interfering with daily functioning, and accompanied by increased anxiety and recurrent depression. Despite resuming sertraline 50 mg, symptoms persisted and gastrointestinal symptoms escalated. Fecal calprotectin was declined and moderately severe UC was confirmed by histopathology. OCD and depressive symptoms persisted despite sertraline, CBT (without ERP), and addition of bupropion XL, with initial insomnia. Current adjunctive psychotherapy was supportive and interpersonal.

Medical history was notable for persistent moderately severe UC with multiple daily bloody stools, hemorrhoids, anal fissure, psoriasis, Hashimoto’s thyroiditis, and migraine. There was no history of liver disease, acute intermittent porphyria, pyruvate carboxylase deficiency, fatty acid oxidation defects, primary carnitine deficiency, palmitoyl transferase I or II deficiency, or carnitine translocase deficiency.

Family psychiatric history revealed OCD, generalized anxiety disorder, post-traumatic stress disorder (PTSD), depression, suicide attempts, and attention deficit hyperactivity disorder (ADHD). Furthermore, family medical history included inflammatory and metabolic disease: Crohn’s, autoimmune conditions, hyperlipidemia, hypertension, and cardiovascular disease.

## Metabolic psychiatry evaluation

She presented to a specialized metabolic psychiatry clinic offering comprehensive psychiatric, dietary, and lifestyle interventions by licensed staff under one roof, described previously (27), seeking to reduce persistent OCD, anxiety, and depression; lose weight; and improve her overall health. Notably, she was not expecting improvement in UC.

A comprehensive psychiatric evaluation revealed obsessive thoughts of harm, persistent anxiety, low mood, amotivation, anhedonia, insomnia, fatigue, poor concentration, increased appetite, weight gain, and poor self-esteem. She spent 2 hours in the bathroom every morning, with cramping and five episodes of bloody diarrhea daily. A review of systems revealed dark undereye circles, snoring, hair loss, hoarse voice, joint pain, brain fog, dry skin, and eczema.

Medications were sertraline 50 mg, bupropion XL 150 mg, mesalamine DR 3.26 g, levothyroxine 100 mcg daily, and topical fluocinonine 0.95% and hydrocortisone 2.5% creams. Physical examination showed diffuse goiter, discrete plaque psoriasis (elbows and knee), and atopic dermatitis in flexor creases.

Nutritional evaluation revealed sweets cravings, sugar bingeing when stressed, and progressive weight gain of 70 lbs/31.8 kg over the past few years. She added sugar to her coffee daily and kept food next to her on her desk at work. A typical day’s food included coffee with sweetened creamer and a bagel, and frequent, intermittent eating including cheese, fruit, sandwiches, chicken nuggets, and mac and cheese, with ice cream or cookies at night and occasional cannabis edibles at bedtime. Previous weight loss efforts spanned an intolerable trial of topiramate, medical weight loss clinics, digital apps, and exercise, with no response. She felt deprived by limiting specific foods, struggled with cravings, and reported a lack of time and motivation. Lifestyle and work were sedentary, and she struggled with household tasks and family activities. She walked her dog 1-2x/week; leisure activities included reading and occasional contact with friends.

Body composition analysis was performed using InBody^®^ 550, a multi-frequency bioelectrical impedance analyzer capable of segmental assessments. Measurements were conducted at baseline and monthly, as per the manufacturer’s guidelines, standing upright with electrodes placed on the hands and feet. Data on body fat percentage and lean body mass were obtained and analyzed using proprietary algorithms provided by InBody^®^.

Laboratories included comprehensive metabolic profile, HbA1c, uric acid, carnitine, calcium, magnesium, zinc, advanced lipid profile with inflammation including HS-CRP, insulin resistance panel with C-peptide, Vitamin B12, methylmalonic acid/homocysteine, Vitamin D 1,25 (OH), CBC, iron, TIBC ferritin, and thyroid panel with TSH and rT3. TSH was 5.4 mIU/ml and LDL 104 mg/dl with a particle number of 1789 and peak size of 219. Furthermore, LDL medium was 427 nmol/L, LDL small 289 nmol/L, HDL large 5919 nmol/L, and apolipoprotein B 92 mg/dl. Despite active symptoms, hsCRP was low at 0.4 mg/L.

Rating scales were administered at baseline and repeated at appropriate intervals.

OCD was assessed monthly by the Yale-Brown Obsessive Compulsive Scale (Y-BOCS) and weekly by the Florida Obsessive Compulsive Inventory (FOCI) and Clinical Global Impression-Improvement/Severity scales (CGI-I/S); these were correlated with a narrative report. UC was assessed using Partial Mayo Score (PMS) and the Ulcerative Colitis Patient-Reported Outcome (UC-PRO) in the absence of repeat fecal calprotectin/colonoscopy. Quality of life was assessed using the Flourishing Scale (FS), Self-Compassion (SC), and Rugged Resilience Measure (RRM) administered monthly and a narrative report. QoL measures specific to OCD and UC were not employed.

Adherence was assessed using daily capillary ketone/glucose testing with a Keto-Mojo^®^ GK+ Blood Glucose and β-Ketone Dual Monitoring System (ketone/glucose correlation coefficients to serum of 0.9927/0.9974) measuring d-beta-hydroxybutyrate (BHB), glucose, and glucose/ketone index (GKI); photojournaling of food intake; and self-report. BHB and GKI were correlated to OCD and UC response/remission.

Metabolic health was assessed by interval bioimpedance measures of weight, lean body mass, percentage body fat, visceral fat levels, and blood pressure. Follow-up labs were declined due to financial constraints.

## Ketogenic diet

A well-formulated, personalized, therapeutic KD, known as KMT, was developed by a ketogenic registered dietitian using a KMT ratio of 1.5:1 (fat: protein + carbohydrates), whole foods, and animal-based protein sources; the macronutrient range was approximately 75% fat, 20% protein, and 5% or less carbohydrate. Seed oils were intentionally eliminated to reduce inflammation (27). KMT incorporated individual food preferences/sensitivities, lifestyle concerns, and work/family schedules. Risks/benefits and alternatives to a ketogenic diet were reviewed and she provided written informed consent for treatment with KMT.

She implemented KMT at home without food delivery services or exogenous ketone supplements, with adequate hydration (water to thirst) and liberal use of unrefined, natural sea salt. Proteins were animal-based due to high bioavailability and superior amino-acid profiles (28).

Commercial ketogenic foods were discouraged as they are often ultra-processed and contain sweeteners, resistant starches, mold inhibitors, and oxidation or leavening agents to prevent off flavors and improve texture. Added sugars, non-nutritive sweeteners, and sweetening agents were eliminated; processed seed oils were intentionally avoided to reduce inflammation (27). Adjunctive nutritional strategies to potentially reduce OCD and anxiety were not employed to reduce variables other than KMT which might contribute to symptomatic improvement, including magnesium supplementation, n-acetylcysteine, caffeine reduction, and use of prebiotics or probiotics.

## Treatment course

She met twice weekly with a ketogenic registered dietitian for 1 month, then once weekly for the duration of treatment. Screening for potential side effects of KMT occurred through daily digital apps and clinical visits.

Citing time constraints, she declined to participate in KMT lifestyle interventions offered by the clinic. These included group meetings, outdoor nature walks, family and friends supports, community building, and monthly book club. She maintained weekly visits with her referring therapist. Psychiatric follow-up visits were scheduled as needed.

Within the first days, she eliminated sugar and sweetened coffee creamer, and ate the following foods on one day in the first week: coffee with cream, scrambled eggs with cheese, leftover steak, cauliflower egg salad with avocado mayo and nuts, chicken, cauliflower mac and cheese with bacon; she replaced bedtime ice cream and cookies with a handful of berries.

Within fifteen days, she lost 9.4 lbs/4.3 kg, and reported significantly improved energy. She felt satiated by meals; cravings and uncontrolled eating had ceased. Her mind felt sharp and clear. However, photojournaling became intermittent, and capillary BHB oscillated with weekly averages of 0.8 mmol/L; despite variation, GKI remained mostly ≤ 6. Although she reported eating “according to plan,” she sometimes forgot to add fats, but responded to encouragement and troubleshooting.

**Figure 1:**
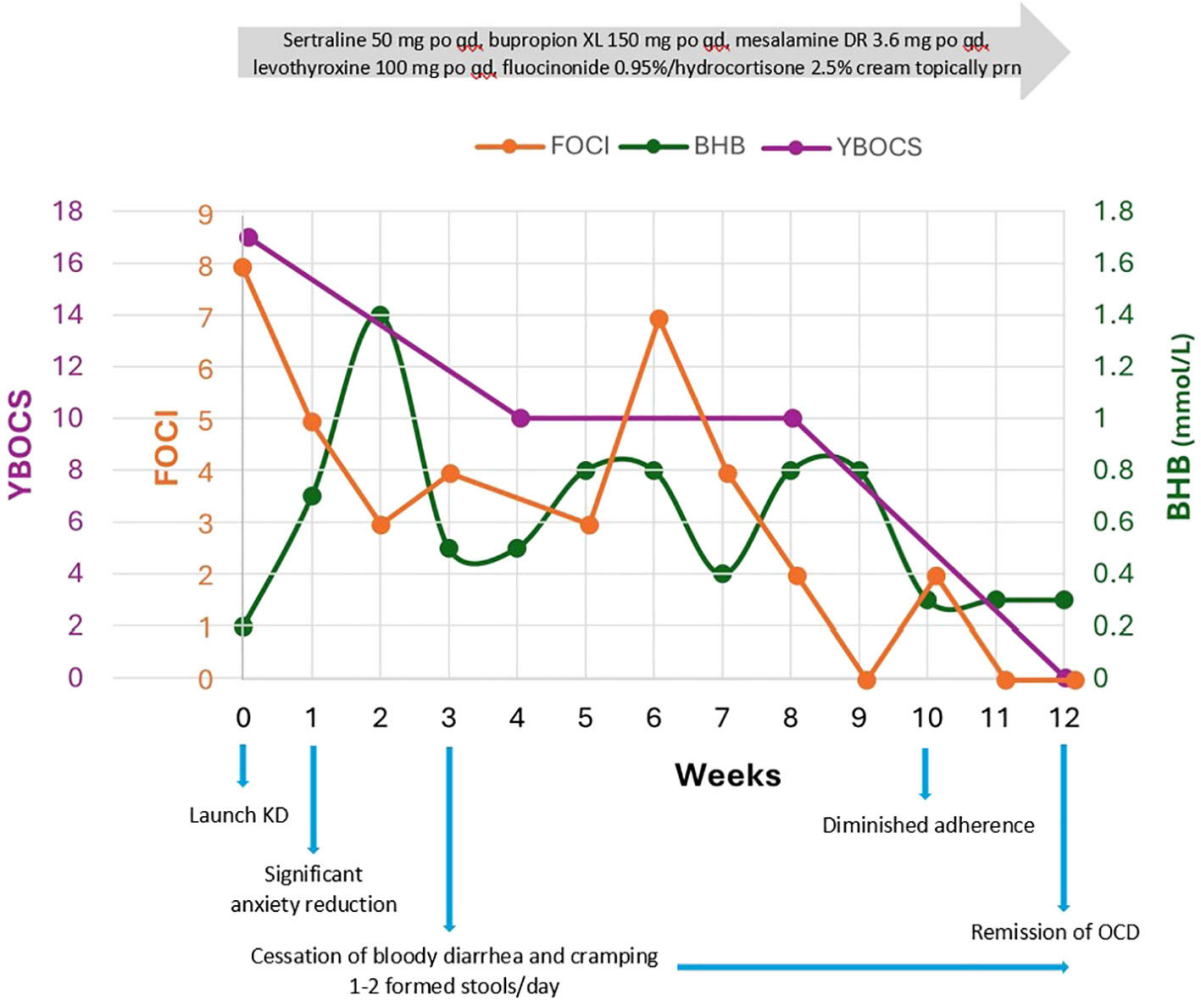
Y-BOCS and FOCI correlated to average weekly capillary BHB (mmol/L) over 12 weeks. Y- BOCS is the gold standard in OCD assessment of symptom breadth, severity, and degree of interference with daily life; it has high internal consistency and convergent validity. FOCI is a brief self-report instrument, including symptom checklist and severity score, shows high correlation with Y-BOCS, and is suitable for tracking treatment progress weekly. FOCI was 0 at 9 weeks and again at 11 and 12 weeks with Y-BOCS 0 and CGI-S 1, signifying robust change and meaningful improvements in everyday life.

Within 3 weeks, all cramping and bloody diarrhea ceased, and stool frequency/form normalized to one stool per day (**Figure 1**). To her surprise, psoriatic plaques cleared. Her energy and concentration were excellent and she handled work stressors exceptionally well. As KMT continued, she worked on “phasing out all high sugar foods in the house.”

She experienced emergent initial insomnia as the only adverse effect of KMT, unresponsive to circadian strategies but responsive to trazodone 50–100 mg. No drug-KMT interactions were identified.

## Remission and quality of life

**Figure 2:**
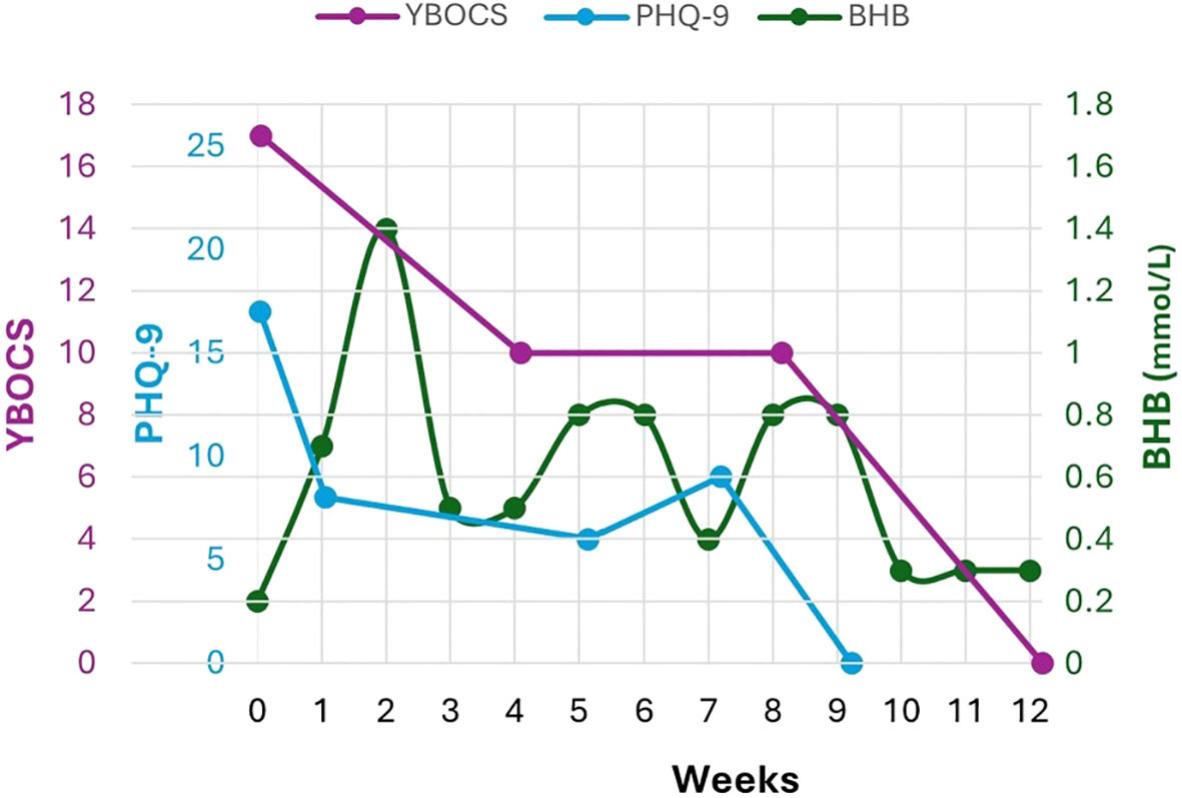
Given the association of comorbid depression in women with increasing disability and doubled health care costs, here we track Y-BOCS and PHQ-9 to average weekly capillary BHB (mmol/L) over 12 weeks of treatment Major depression remitted before OCD, at 9 weeks (PHQ-9=0). Despite ketone fluctuations, GKI remained mostly ≤ 6.0 with few exceptions.

OCD remitted (FOCI 0) within 9 weeks and she met the full remission criteria for OCD (Y-BOCS 0, CGI-S 1) by 12 weeks, capturing meaningful improvements in everyday life (**Figure 1**). Major depression also remitted within 9 weeks (PHQ-9 decreased from 17 to 0) (**Figure 2**). The Yale Food Addictions Scale (YFAS 2.0 ) decreased from 4 to 0 within 4 weeks, corresponding with early cessation of sugar cravings and binges.

UC showed clinical remission in 3 weeks (Partial Mayo Score 7 to 0, UC-PRO 5 to 0), and psoriatic plaques cleared within 8 weeks.

Metabolic health improved significantly over 12 weeks: she experienced a 9.4% loss of body weight (21.5 lbs/9.8 kg) and 3.2% decrease in body fat percentage (45.3 to 42.1%); segmental fat analysis showed truncal fat loss of 7.5 lbs/3.4 kg and decrease in visceral fat levels from 21 to 18. Autoimmune conditions improved with a clearing of psoriatic plaque and normalization of TSH; complete remission of Hashimoto’s could not be established as she declined retesting of thyroid antibodies due to financial constraints.

**Figure 3:**
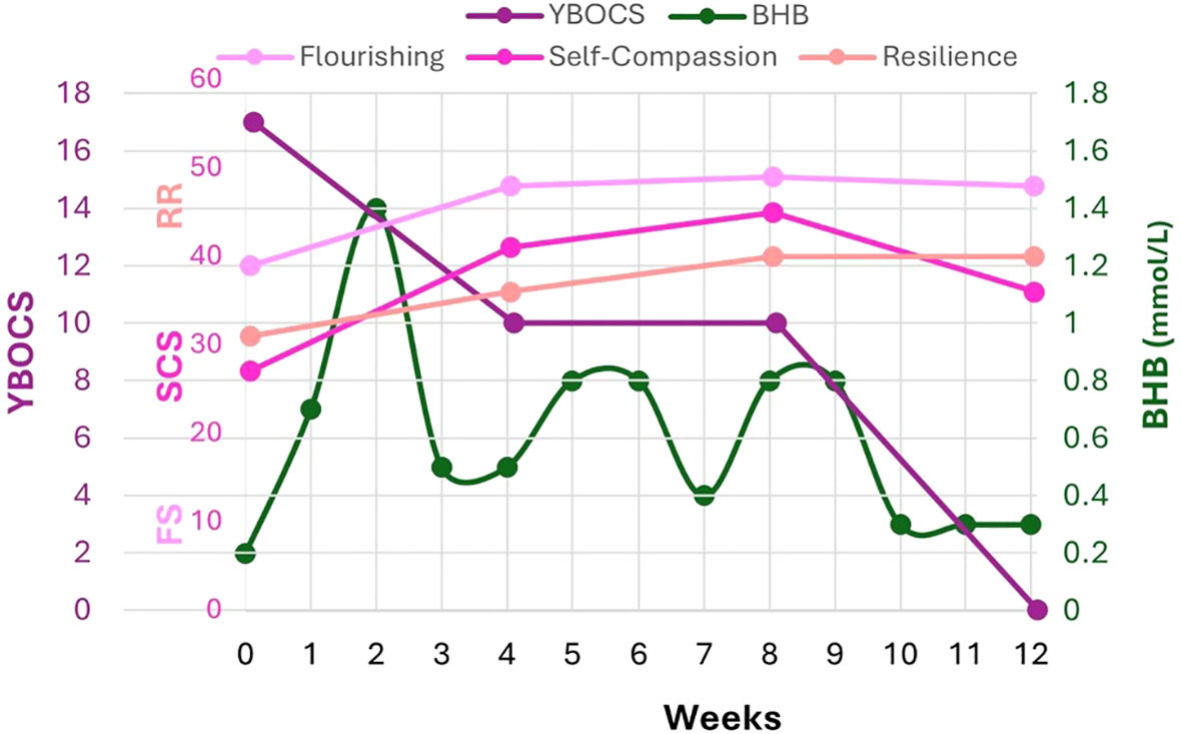
FS, SCS, RRM validated scales correlated to Y-BOCS and capillary BHB (mmol/L). FS focuses on psychological flourishing and captures strengths like optimism, purpose, self-esteem, and relationships. SCS measures positive and negative correlates of self-compassion; self-kindness, mindfulness, and shared humanity have been correlated with positive health outcomes; self-judgment, isolation and overidentification with negativity are correlated with depression, anxiety and poor health outcomes. RRM focuses on key attributes associated with internal resilience and coping with adversity. In the absence of an established Minimal Clinically Important Difference (MCID) for FS, SCS, and RRM, half of a standard deviation in scoring is conventionally used to assess meaningful or clinically significant change. Thus, the following changes are considered clinically significant: FS 3- 4 points, SCS 0.25-0.30 points, RRM 4-5 points. By 12 weeks, FS increased 9 points, SCS 6 points, RRM 4-5 points.

QoL improved according to narrative reports, and the increases in FS, SCS, and RRM were significantly higher than the meaningful threshold, ranging from 2-fold (RRM 31-40) to 3-fold (FS 39-48) to 20-fold (SCS 27-36) above baseline (**Figure 3**).

She expressed great satisfaction with her outcomes but declined additional follow-up including laboratories and fecal calprotectin due to financial constraints. An upcoming colonoscopy was postponed.

## Patient perspectives

Early: “I’m surprised that this is not harder, and that I’ve lost so so much weight so easily where I couldn’t ever before. The salt is definitely making a difference—I’ve been low salt my whole life, so this is really a big change. I’m putting salt in my water.” Mid to late: “I am so much better overall. When I got a GI bug, [my gut] was OK after two days, and that never would have happened before—I’d be symptomatic for weeks–this is so different it’s incredible.” Her perspective was positive: she was struck by how easily she adapted to KMT, lost weight, and lost percentage body fat after years of failed attempts, and then, with continued ketogenic treatment, found widespread improvements in her mental health, gut health, and overall well-being.

## Discussion

Despite emerging research exploring the efficacy of ketogenic diets in major depression, bipolar disorder, and schizophrenia (26, 29–34), and emerging interest in ketogenic diets as a transdiagnostic treatment for neuropsychiatric disorders (35, 36), there is little data regarding KMT treatment of OCD. Obsessive thoughts, without meeting the full criteria for OCD, are described in autism and anorexia (37, 38). Reduced obsessive thoughts have been reported in children with autism and epilepsy treated with KMT (37) and in a single case and subsequent small open trial of adult women treated with KMT followed by ketamine infusions (38). Similarly, there is little data describing KMT treatment and response specifically for UC; one case series described self-reported improvement of 10 individuals with varied IBD who adopted a carnivore-ketogenic diet (39).

This case is unique because KD/KMT has not previously been reported for the treatment of OCD and here, within 12 weeks, this individual experienced not only complete remission of a 5-year history of distressing harm OCD embedded in a 25-year OCD history but clinical remission of a 5-year cascade of inflammatory and autoimmune disorders which began with UC, as well as improvements in metabolic health with significant loss of body weight and percentage body fat. Contemporaneous remission of OCD and UC is not only rare, it has never been previously correlated to a single therapeutic intervention.

The intertwining course of her symptoms leading to KMT suggests the evolution of complex bidirectional inflammatory processes linking microbiome, gut, systemic, and neuroinflammation, potentially accounting for the escalation of OCD intensity and new harm obsessions following the onset of UC.

UC and OCD are both associated with gut microbiome alterations (14, 40) and it is possible that in the setting of longstanding OCD, the emergence of UC triggered an inflammatory cascade resulting in neuroinflammation, dramatic worsening of OCD, and a bidirectional feedback loop of gut-systemic-neuro inflammation which impaired insulin signaling and resulted in carbohydrate craving, progressive obesity, and central adiposity.

We propose contemporaneous clinical remission of OCD and an inflammatory bowel disease such as UC may arise because bidirectional inflammation can begin with gut dysbiosis and disruption of epithelial cell tight junctions in UC, triggering the overproduction of lipopolysaccharides (LPS) and short-chain fatty acids (SCFA). This can simultaneously stimulate the vagus nerve and activate the NLRP3 inflammasome in peripheral immune cells, prompting the release of pro-inflammatory cytokines; both compromise the blood-brain barrier (BBB) (40, 41). Systemic inflammatory factors can then enter the CNS, activating microglial NLRP3 inflammasomes. The mechanisms are complex but elegant: microglia are densely expressed throughout the CSTC circuit. When activated, they amplify and stimulate continued neuroinflammation by releasing cytokines, ROS, and chemokines (42). Due to microglial density, the CTSC may be particularly susceptible to local neuroinflammation and its secondary effects on neurotransmitter expression and function.

This is critical because the etiology of OCD is increasingly considered to involve neuroinflammation.

As a single intervention, KD may directly reduce OCD symptoms in the CTSC circuit through the same mechanisms by which it can heal the gut and suppress systemic inflammation (43). KDs have been shown to reduce the severity of colitis in 1-month murine studies by alleviating inflammation and maintaining tight junction protein levels (44). In addition, BHB directly inhibits TLR4 signaling to mitigate neuroinflammation, modulates SCFA production, inhibits NLRP3 inflammasome activation of microglia, and reduces the release of IL-1B and IL-18 (45, 46), inflammatory cytokines which are key drivers of OCD-related neuroinflammation; furthermore, acetoacetate suppresses glutamate release (25, 42, 47).

This case report is limited by selection and reporting bias, as this patient expressed interest in KMT after years of inadequate treatment response. The absence of a control group prevents the establishment of a causal relationship between KMT and remission of OCD and UC. Fecal calprotectin and inflammatory biomarkers beyond initial Hs-CRP were not obtained. Some rating scales and capillary BHB measures were missing, which may impact the accuracy of weekly average BHB and the exact correlation to symptomatic improvement. Intermittent lack of adequate dietary fat and non-adherence likely contributed to BHB oscillation. The lack of consistent nutritional ketosis may have impacted time to remission; the lack of clinical and laboratory follow-up impedes a complete assessment of metabolic health. Participation in varied lifestyle interventions may have improved adherence or resulted in a more rapid response; however, non-participation removes those potential confounders to the biological effects of KMT.

Although this case illustrates the use of a therapeutic ketogenic diet as one intervention to target two seemingly diverse conditions, OCD and IBDs such as UC are bidirectional and linked by inflammatory mechanisms through the microbiota-gut-brain-axis. As the field of metabolic psychiatry advances, further research is needed to investigate the therapeutic potential of ketogenic diets in OCD and related disorders and to explore their effects on the microbiome-gut-brain axis, neuroinflammation, CSTC activity, neurotransmitter modulation, and insulin signaling, as well as their roles in enhancing innate immunity, modulating gene expression, and restoring metabolic health.

## Data Availability

The original contributions presented in the study are included in the article. Further inquiries can be directed to the corresponding author.
